# Impact of Dog’s Age and Breed on Dog Owner’s Physical Activity: A German Longitudinal Study

**DOI:** 10.3390/ani12101314

**Published:** 2022-05-20

**Authors:** Benedikt Hielscher-Zdzieblik, Ingo Froboese, James Serpell, Udo Gansloßer

**Affiliations:** 1 Institute of Movement Therapy and Movement-Oriented Prevention and Rehabilitation, German Sport University Cologne, 50933 Cologne, Germany; i.froboese@dshs-koeln.de; 2 Institute of Zoology and Evolutionary Research with Phyletic Museum, Friedrich Schiller University Jena, 07743 Jena, Germany; udo@ganslosser.de; 3 Department of Clinical Studies VHUP, School of Veterinary Medicine, University of Pennsylvania, Philadelphia, PA 19104, USA; serpell@vet.upenn.edu

**Keywords:** dog-related physical activity, dog walking, longitudinal, agility, obedience, activity types

## Abstract

**Simple Summary:**

Dog ownership has been linked to physical activity of the owners in several countries. Physical activity is also affected by age, size and energy level, as perceived by the owners, of the dogs. Earlier studies were mostly cross-sectional, which does not allow causal conclusions. This study aimed to find differences and changes in the physical activity behavior of owners of ten different dog breeds that were selected based on their size and energy level. Nine dog breed groups were used and owners filled out an online physical activity questionnaire once per year for three years. The results show that dog owners’ total and dog-related physical activity as well as their leisure time and dog walking decreased over time. Owners of the dog breed groups differed in all physical activity variables. If only participants who completed the study were analyzed, no changes in any physical activity variable were found. At baseline, owners of different dog breeds differed in the types of reported dog-related activities. Overall, the results indicate that physical activity behavior in dog owners is stable over time. However, no clear pattern could be found based on the age, size and energy level of the dogs.

**Abstract:**

Dog ownership contributes positively to physical activity (PA). The impact of different dog breeds and age on PA is less investigated in longitudinal studies. This study aimed to evaluate PA changes in dog owners as their dogs’ ages increased and to explore whether there are differences in PA between owners of different breeds over a three-year period. Owners of different dog breeds were categorized into nine groups according to the perceived energy level and size of the breed. PA was monitored using an online questionnaire for three consecutive years. Linear mixed models (LMM) showed a small, but significant decrease in total PA, leisure time walking, dog-related PA and dog walking over three years. No decreases were found if only participants who attended at all time points were included. In all LMM analyses, a significant relationship between the dog breed and the outcomes of PA were shown. At baseline, dog owners performed different types of activities depending on their dog breed. In conclusion, owners of different dog breeds differ in their types of PA. The study emphasizes that age, size and energy level of the dog does not per se have an impact on dog owners PA.

## 1. Introduction

Recent studies have shown that dog ownership is associated with increased physical activity (PA) in Australia [1], Canada [2], the Czech Republic [3,4], Finland [5], Germany [6], Japan [7], the United Kingdom [8,9,10,11,12,13,14], South Korea [15] and the USA [16]. Dog owners have also a higher number of steps per day on average than non-dog owners [4,8]. Much of the PA of dog owners consists of dog walking [6,17,18]. Most previous studies of the relationship between dog ownership and PA were cross-sectional, and only a few longitudinal studies give information about the causal relationship between dog ownership and owners’ PA. An early study from the UK found significant increases in PA after acquiring a dog [14]. An Australian study showed that dog acquisition leads to an increase in dog walking but not total PA [19]. Two other investigations detected an initial increase in daily steps after dog acquisition that was diminished at a second follow-up period [20,21]. Therefore, it could be concluded that dog acquisition might increase PA in prospective dog owners.

Physical inactivity is associated with poor health and an increased mortality risk [22,23,24,25,26]. It has been documented that dog ownership also correlates inversely with the risk of several diseases and mortality [27] and that dog owners have a better cardiovascular condition than people who do not own any pets [3]. However, the authors of a recent meta-analysis identified only a non-significant reduction in the mortality risk for dog owners and, therefore, advocate treating these previous results with caution [28].

It has been shown that the size of a dog [29,30,31,32,33,34,35] and their owner-perceived energy level [31,36] are positively associated with dog walking. Moreover, it has been shown that the average energy levels of dogs, as perceived by their owners, vary between breeds and breed groups [37,38].

The age and the health status of the dog have also been shown to be associated with dog walking [35,36,39,40,41,42]. The probability of being walked is smaller for older dogs and dogs with a poorer health status as compared to younger and healthier dogs [35,36]. Furthermore, dog walking behavior changes as dogs develop health problems [41]. However, since the aging process of dogs of different sizes varies significantly [43], dogs of different sizes might influence dog owners PA differently with increasing age.

The aim of this study was, therefore, to investigate differences at baseline and changes over time in the PA of dog owners of different dog breeds. It was hypothesized that owners of smaller dogs with a lower energy level would be less physically active overall. It was expected that the PA of dog owners would decline over the years as the dogs aged. Furthermore, if a dog died it was expected that the PA behavior of the owner would decline, if there was no dog left in the household.

## 2. Materials and Methods

### 2.1. Participants

Study participants were required to be at least 18 years old and to have at least one dog of ten specified dog breeds at the time of recruitment. Participants that owned more than one of the selected breeds were categorized as a separate group. In order to achieve a sufficiently large sample, an average number of 500 puppies registered in the German subsection of the FCI (VDH) per year in the period from 2010 to 2014 [44] was used as a criterion. The dog breeds were then selected based on two criteria:

First, the dog breeds were divided into categories based on the height at the withers as specified in the Féderation Cynologique International (FCI) standard (small ≤ 40 cm; medium = 40–59 cm; large ≥ 60 cm). If the breed standard recorded a size that exceeded the defined size limits, the breed was placed in the larger category. Then, the energy level was evaluated, as measured by the Canine Behavioral Assessment and Research Questionnaire (C-BARQ). We used data from the C-BARQ project at the University of Pennsylvania (https://vetapps.vet.upenn.edu/cbarq/, assessed on 15 March 2022) to estimate the energy levels of the different breeds. Participants in the current study did not complete the C-BARQ questionnaire.

The C-BARQ is an online survey that allows owners to evaluate the behavior of individual dogs [45]. Energy level is one of 14 behavioral dimensions evaluated by the C-BARQ and it consists of two questionnaire items: “playful, puppyish, boisterous” and “active, energetic, always on the go” (Serpell and Duffy, 2014, p. 48) [37]. Both items are scored on five-point scales from 0 to 4, with a higher score indicating that the behavior is exhibited more frequently [37,46]. The score for energy level represents the average of the scores for these two items.

The selected breeds are:

1. Cavalier King Charles Spaniel (CKCS) [47] (small size, low energy)
2. West Highland White Terrier (WHWT) [48] (small size, medium energy)
3. Jack Russell Terrier (JRT) [49] (small size, high energy)
4. Parson Russell Terrier (PRT) [50] (small size, high energy)
5. Whippet (WHIP) [51] (medium size, low energy)
6. Labrador Retriever (LAB) [52] (medium size, medium energy)
7. Border Collie (BC) [53] (medium size, high energy)
8. Bernese Mountain Dog (BMD) [54] (large size, low energy)
9. Rottweiler (ROTT) [55] (large size, medium energy)
10. Belgian Shepherd Dog (BSD) [56] (large size, high energy)

To take part in the study, participants needed to be able to understand, read and write in German. They could own a maximum of 5 dogs. Participants were excluded if they reported not owning a purebred dog of the selected breeds or if they reported that they did not complete the questionnaire accurately. Furthermore, they were excluded if they had help from another person, because this might bias the results, e.g., because of social desirability [57].

Participants were recruited using groups that focused on the selected dog breeds on social media. A description of the study was posted alongside a link that led to the online questionnaire. Permission was obtained from the group administrators before the link was posted. For each dog breed two to four groups agreed to the posting. The number of group members per group varied between 203 and 7778. Further, several sub-organizations of the German Kennel Club (VDH) that care for the welfare of the dog breeds were contacted. Some of these associations published an appeal in their club newspapers or contacted their members directly.

### 2.2. Measurements

A 15-min online questionnaire was used. By answering question 1 participants gave informed consent actively (see Supplementary Materials). Participants self-reported sociodemographic and anthropometric data. Body mass index (BMI) was calculated from self-reported height and weight as kg/m^2^. Information on the age, sex, sizes (measured standing at the withers in cm), weight in kg, neuter status and breed of each dog was also provided. Participants completed the questionnaire only once per time point.

Participants also completed the Physical Activity, Exercise and Sport Questionnaire (Bewegungs- and Sportaktivitätsfragebogen [BSA-F], Version 1.0) by Fuchs et al. [58]. An English translation of the BSA-F is available for download at the University of Freiburg (https://www.sport.uni-freiburg.de/de/institut/psychologie/messinstrumente/Messung_der_Sport_und_Bewegungsaktivitaet, assessed on 15 March 2022) [59]. It measures PA in minutes per week over the previous four weeks. The BSA-F was validated by Fuchs et al. and correlates with physical fitness [58].

In addition to the BSA-F, questions were included that specifically asked about PA performed together with the dog (dog-related PA). The questions were based on the BSA-F. Participants were asked about the frequency and duration they walked their dog or rode a bicycle with their dog. Finally, they could report five other dog-related PAs in a semi-open question design. All PA related outcomes were calculated as hours per week (h/week). This approach has been used in earlier studies [6,17].

### 2.3. Procedure

Participants were recruited from 1 August 2017 until 31 July 2018. At baseline (T0) they completed the questionnaire. Participants were asked to create an individual code from their initials and their date of birth in order to enable the data to be assigned to the different points in time.

The questionnaires were made available on the data survey tool https://www.soscisurvey.de (assessed on 15 March 2022). Soscisurvey is a German company that complies with the German and European data protection guidelines [60].

At the first (T1), second (T3) and third (T4) year of follow-up participants received three e-mails within 20 days that reminded them to participate in the study. In addition to the BSA-F, they were asked to report any changes in dog ownership status. In particular, they were asked whether any of the dogs had died. Not completing the questionnaire at follow-up dates was interpreted as withdrawal from the study. The data collection ended two weeks after the last participant received the last reminder at T3.

### 2.4. Statistical Analysis

Unless otherwise specified, descriptive values are reported as mean (*M*) ± standard deviation (*SD*). Outliers were identified using the mean values ± 3 *SD*s. Outliers outside this range were winsorized and changed to the calculated maximum or minimum value.

Baseline values of all parameters were compared between the study groups to show accordance for demographic and anamnestic parameters. For all tests of the descriptive analysis: In case of normally distributed continuous data (examined using a Shapiro Wilk test) *t*-tests were used for group comparison. Non-normally distributed continuous data, and ordinal data were tested via Kruskal Wallis tests. Categorical data were tested by *χ*^2^-tests.

Changes over time were analyzed using a linear mixed model (LMM). A maximum likelihood approach was used. Linear, quadratic and cubic time trends were tested as described by Shek and Ma [61]. Breed groups were used as predictors with owners of CKCS being the reference group, since owners of the smallest breed with the lowest energy levels were hypothesized to be least active. A random intercept was used for subjects. All other variables were defined as fixed. The time points were nested within individuals. The model was built using a step-by-step approach, adding one predictor at a time. First, the time trends were added one by one. If an added variable (e.g., quadratic trend) did not improve the model, the next stage was discarded (e.g., cubic trend). The best models were identified using the −2 log-likelihoods of the separate models and *χ*^2^-tests as recommended by Field and Tabachnik and Fidell [62,63].

Additionally, a completer analysis was performed. For this purpose, only participants who had completed the whole study were examined using a repeated measures ANOVA. Due to the small sample sizes only within group analysis were performed.

If participants stated at one point in time that they owned several of the selected breeds and at another point in time only one of the selected breeds, only the latter was retained in the LMM. Participants that owned more than one of the specified breeds at one point in time were excluded from the ANOVA because only the effects of the individual dog breeds should be examined.

The level of statistical significance was set at α = 0.05 in all tests. All analyses were performed using IBM SPSS Statistics Version 27.

## 3. Results

### 3.1. Classification of Dog Breeds

JRT and PRT were merged together to form a single group in order to ensure a sufficiently large sample size. They do not differ in their energy level as evaluated by the C-BARQ (*t* = 1.18, *df* = 413, *p* = 0.239).

Overall, significant differences in energy level were found within the groups of small (CKCS, WHWT, JRT/PRT) (*F* (2, 665) = 19.03, *p* < 0.001), medium (WHIP, LAB, BC) (*F* (2, 2824) = 29.11, *p* < 0.001) and large (BERN, ROTT, BSD) (*F* (2, 934) = 54.45, *p* < 0.001) dog breeds. Linear trends were analyzed and shown to be significant (*F*_small_ (1, 665) = 34.39, *p*_small_ < 0.001; *F*_medium_ (1, 2824) = 51.12, *p*_medium_ < 0.001; *F*_large_ (1, 934) = 92.45, *p*_large_ < 0.001). Compared to WHWT (2.01 ± 0.97), CKCS (1.78 ± 1.01) had a lower and JRT/PRT (2.37 ± 1.07) had a higher energy level. In contrast to LAB (2.02 ± 1.08), WHIP (1.63 ± 0.98) exhibited a lower energy level, while BC exhibited higher energy levels (2.25 ± 1.05). Taking the energy levels of ROTT (1.97 ± 1.06) as reference, the energy levels of BERN (1.78 ± 0.96) were lower and of BSD (2.80 ± 0.99) were higher.

### 3.2. Baseline Characteristics of the Study Population and Dog Breeds

**Figure 1:**
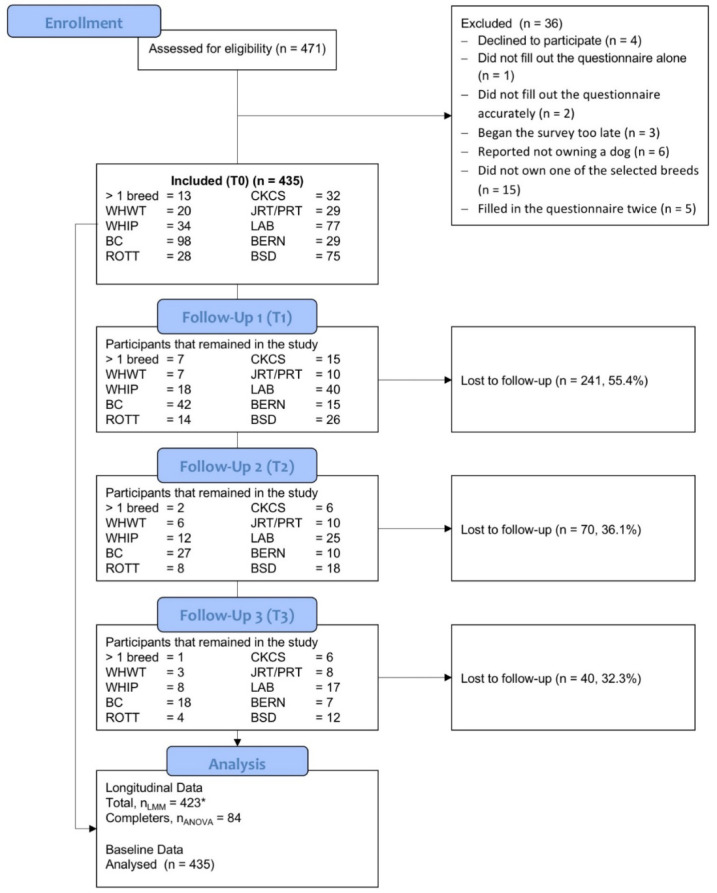
Flow chart of participants over the course of the study. * 12 participants reported owning more than one of the specified dog breeds at all times of the study and were, therefore, excluded from the LMM. BC, Border Collie; BERN, Bernese Mountain Dog; BSD, Belgian Shepherd Dog; CKCS, Cavalier King Charles Spaniel; JRT/PRT, Jack and Parson Russell Terrier; LAB, Labrador Retriever; PA, physical activity; ROTT, Rottweiler; WHIP, Whippet; WHWT, West Highland White Terrier.

At T0 435 dog owner participated, of which 84 completed the study. Thus, the dropout rate was 80.7%. For a more detailed description see Figure 1. The number of participants per breed at T0 varied from 20 (WHWT) to 98 (BC).

An overview of the statistically significant differences of the sociodemographic and anthropometric variables at baseline is summarized in Table 1. There were differences in the distribution in smoking status (*χ*^2^ = 20.47, *df* = 9, *p* = 0.015), educational attainment (*H* = 43.41, *df* = 9, *p* < 0.001), income in € (*H* = 25.40, *df* = 9, *p* = 0.003), children under 18 years of age living in the household (*χ*^2^ = 16.92, *df* = 9, *p* = 0.050), age of the participant (*H* = 27.71, *df* = 9, *p* < 0.001) and the number of dogs living in the household (*H* = 37.36, *df* = 9, *p* < 0.001). No differences in the distribution between the dog owner groups were detected in terms of gender (*χ*^2^ = 10.92, *df* = 9, *p* = 0.282), completer status (*χ*^2^ = 4.76, *df* = 9, *p* = 0.855), relationship status (*χ*^2^ = 7.89, *df* = 9, *p* = 0.545), employment status (*χ*^2^ = 23.04, *df* = 9, *p* = 0.189), size of hometown (*H* = 6.49, *df* = 9, *p* = 0.690), people over the age of 59 years living in the household (*χ*^2^ = 4.32, *df* = 9, *p* = 0.889), garden ownership (*χ*^2^ = 14.76, *df* = 9, *p* = 0.098), chronic diseases of participants (*χ*^2^ = 8.94, *df* = 9, *p* = 0.443) or the BMI (*H* = 16.55, *df* = 9, *p* = 0.056). On average, participants engaged in 26.6 ± 15.8 h/week total PA, 14.7 ± 8.5 h/week dog-related PA, 12.0 ± 7.2 h/week leisure time walking and 11.3 ± 6.9 h/week dog walking at baseline.

Differences between the dogs of different breeds are displayed in Table 2.

### 3.3. Baseline Comparison of PA

**Figure 2:**
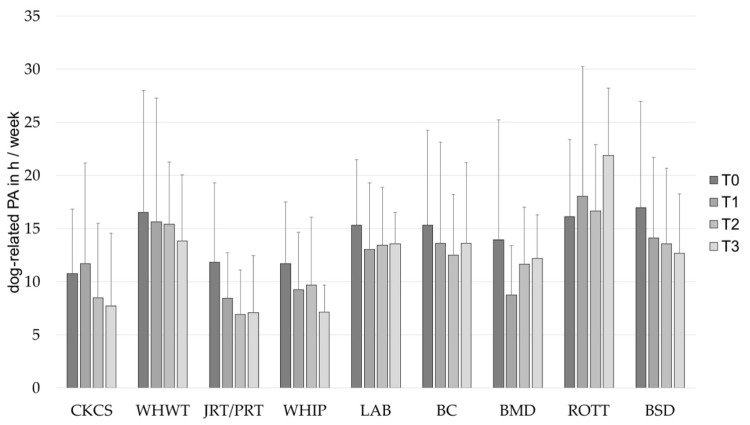
Dog-related PA in h/week (mean ± *SD*) of owners of different dog breeds at baseline (T0), after 1 (T1), 2 (T2) and 3 (T3) years. BC, Border Collie; BERN, Bernese Mountain Dog; BSD, Belgian Shepherd Dog; CKCS, Cavalier King Charles Spaniel; JRT/PRT, Jack and Parson Russell Terrier; LAB, Labrador Retriever; PA, physical activity; ROTT, Rottweiler; WHIP, Whippet; WHWT, West Highland White Terrier.

**Figure 3:**
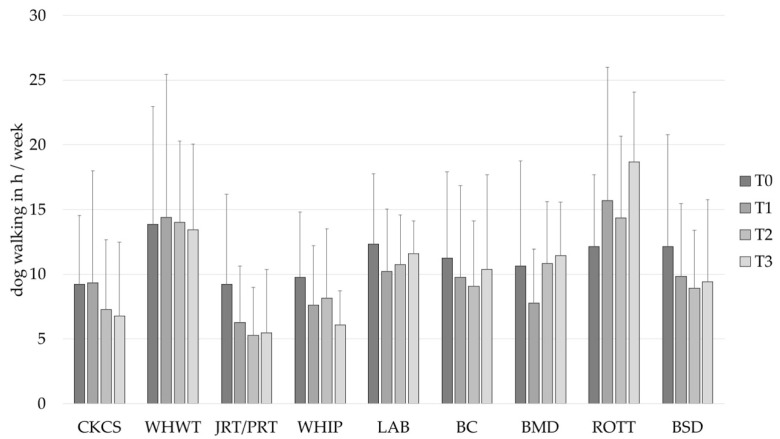
Dog walking in h/week (mean ± *SD*) of owners of different dog breeds at baseline (T0), after 1 (T1), 2 (T2) and 3 (T3) years. BC, Border Collie; BERN, Bernese Mountain Dog; BSD, Belgian Shepherd Dog; CKCS, Cavalier King Charles Spaniel; JRT/PRT, Jack and Parson Russell Terrier; LAB, Labrador Retriever; ROTT, Rottweiler; WHIP, Whippet; WHWT, West Highland White Terrier.

**Figure 4:**
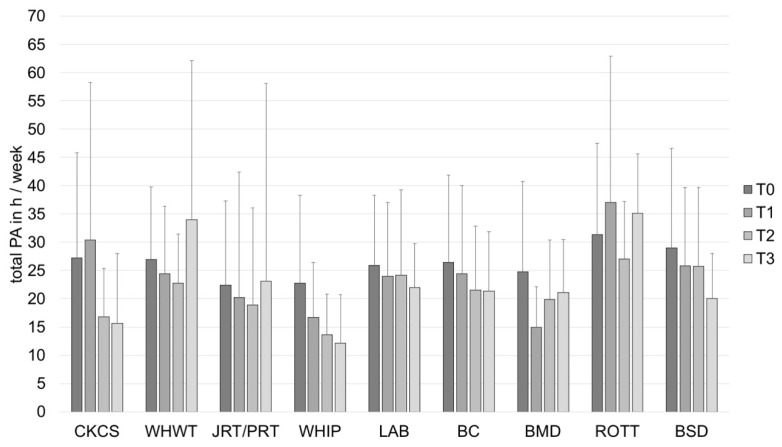
Total PA in h/week (mean ± *SD*) of owners of different dog breeds at baseline (T0), after 1 (T1), 2 (T2) and 3 (T3) years. BC, Border Collie; BERN, Bernese Mountain Dog; BSD, Belgian Shepherd Dog; CKCS, Cavalier King Charles Spaniel; JRT/PRT, Jack and Parson Russell Terrier; LAB, Labrador Retriever; PA, physical activity; ROTT, Rottweiler; WHIP, Whippet; WHWT, West Highland White Terrier.

**Figure 5:**
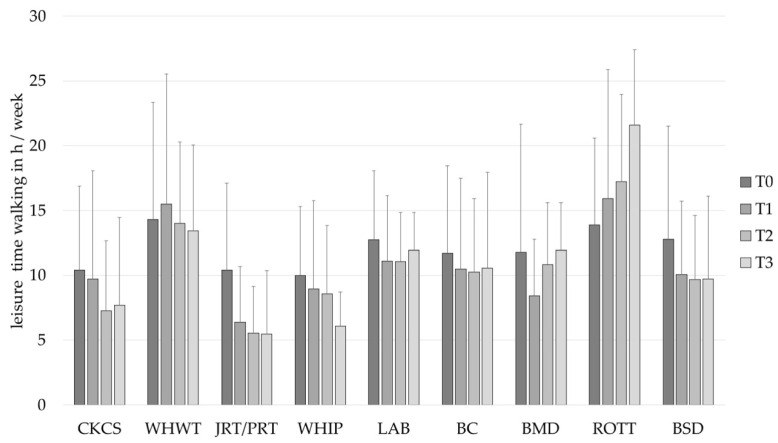
Leisure time walking in h/week (mean ± *SD*) of owners of different dog breeds at baseline (T0), after 1 (T1), 2 (T2) and 3 (T3) years. BC, Border Collie; BERN, Bernese Mountain Dog; BSD, Belgian Shepherd Dog; CKCS, Cavalier King Charles Spaniel; JRT/PRT, Jack and Parson Russell Terrier; LAB, Labrador Retriever; ROTT, Rottweiler; WHIP, Whippet; WHWT, West Highland White Terrier.

At baseline, the outcomes of PA were analyzed with all participants who owned one of the specified dog breeds, but without the owners that reported having more than one of the specified breeds. Statistically significant differences in dog-related PA (*H* (8) = 26.99, *p* < 0.001, Figure 2) and dog walking (*H* (8) = 16.49, *p* = 0.036, Figure 3) were found between the owners of the specified dog breeds. Differences in total PA (*H* (8) = 13.78, *p* = 0.088, Figure 4) and leisure time walking (*H* (8) = 15.46, *p* = 0.051, Figure 5) did not reach statistical significance. The following dog-related activities or activities that could be performed with a dog were reported most frequently by the participants: bicycle riding (n = 108), ball work (activities that were indicated as using a ball with the dog, like “ball play” or “fetching the ball”) (n = 75), jogging (n = 72), (rally)obedience (activities in which the dog’s obedience is practiced on a course) (n = 72) and agility (n = 67). Statistically significant differences between the owners of the selected dog breeds were identified in ball work, (rally)obedience and agility (Table 3).

### 3.4. Changes of PA over Time

The results of total PA show statistically significant variability across participants, *Var*(*u_0j_*) = 149.07, standard error (*SE*) = 15.29, *Wald Z* = 9.75, *p* < 0.001. There is a statistically significant linear decrease of total PA over time, *F* (1, 498.63) = 6.85, *p* = 0.009. The breed groups were found to differ from one another, *F* (8, 406.28) = 2.21, *p* = 0.026. However, no significant differences were found if the individual estimates of dog breeds were compared to CKCS (Figure 4, Table 4).

Dog-related PA shows statistically significant variability among individuals, *Var*(*u_0j_*) = 49.37, *SE* = 4.58, *Wald Z* = 10.79, *p* < 0.001. The results demonstrate a statistically significant linear decrease over time, *F* (1, 440.56) = 12.58, *p* < 0.001. Additionally, the owners of the different dog breeds were found to differ statistically significantly from each other, *F* (8, 400.20) = 3.46, *p* = 0.001. It was found that owners of WHWT, LAB, BC, ROTT and BSD engage in significantly more dog-related PA than owner of CKCS (Figure 2, Table 4).

Leisure time walking was identified to differ statistically significantly between individuals, *Var*(*u_0j_*) = 34.62, *SE* = 3.27, *Wald Z* = 10.59, *p* < 0.001. A statistically significant decrease of leisure time walking was identified over time, *F* (1, 446.88) = 3.87, *p* = 0.050. Additionally, the interaction term between the linear time trend and the breed groups was found to be statistically significant, *F* (8, 444.16) = 2.36, *p* = 0.017, indicating that the leisure time walking changes over time, depending on the dog breed. However, no effect was found for the breed groups itself, *F* (8, 461.78) = 1.50, *p* = 0.153. The interaction term demonstrates that the owners of ROTT increase their leisure time walking in comparison to owners of CKCS (Figure 5, Table 4).

Statistically significant individual differences were found in the participants in dog-walking, *Var*(*u_0j_*) = 31.46, *SE* = 3.03, *Wald Z* = 10.39, *p* < 0.001. A negative linear trend was identified over time, *F* (1, 425.62) = 7.77, *p* = 0.006. However, a quadratic increase over time was also found to be statistically significant, *F* (1, 396.47) = 4.41, *p* = 0.036. This suggests that there is a steeper decrease in the beginning of the study. Further, the breed groups were found to differ significantly, *F* (8, 396.09) = 2.23, *p* = 0.025. It was found that owners of WHWT, LAB and ROTT engaged in significantly more dog walking than owners of CKCS (Figure 3, Table 4).

### 3.5. Changes of PA in the Completers Population

To identify if those who completed all questionnaires did not differ from those who completed questionnaires only at some time points, a completers analysis was carried out. The significant differences of completers and non-completers in the sociodemographic variables are shown in Table 5. There were differences between completers and non-completers in gender (*χ*^2^ = 5.15, *df* = 1, *p* = 0.024), smoking status (*χ*^2^ = 6.59, *df* = 1, *p* = 0.012), educational attainment (*U* = 11,062, *z* = (3.70, *p* < 0.001), employment status (*χ*^2^ = 9.40, *df* = 2, *p* = 0.009), income in € (*U* = 13,167, *z* = 3.59, *p* < 0.001) and chronic diseases of the participants (*χ*^2^ = 8.93, *df* = 1, *p* = 0.002). No differences were detected in breed that the participant owns (*χ*^2^ = 4.76, *df* = 9, *p* = 0.855), relationship status (*χ*^2^ = 0.07, *df* = 1, *p* = 0.787), size of hometown (*U* = 13,957, *z* = −0.79, *p* = 0.428), garden ownership (*χ*^2^ = 1.91, *df* = 1, *p* = 0.205), children under the age of 18 years (*χ*^2^ = 0.01, *df* = 1, *p* = 1.000) or adults over the age of 59 years (*χ*^2^ = 0.10, *df* = 1, *p* = 0.867) living in the household, age of the participant (*t* = (1.14, *df* = 139, *p* = 0.257), BMI (*t* = 0.02, *df* = 423, *p* = 983), number of dogs in the household (*t* = 1.18, *df* = 140.2, *p* = 0.242), age of the dog (*t* = −0.42, *df* = 886, *p* = 0.676), size of the dog (*t* = −0.05, *df* = 891, *p* = 0.961) or weight of the dog (*t* = 0.36, *df* = 893, *p* = 0.719).

Significant differences in total PA and dog-related PA were found between completers and non-completers at T0 and in total PA at T1. No other significant differences appeared in total PA and dog-related PA. In leisure time walking and dog walking no significant differences were found between completers and non-completers at any time point (Table 6). However, non-completers scored higher in leisure time and dog walking at T0 and T1, but lower at T2 than completers.

If only completers were analyzed using a repeated measures ANOVA, no changes in any of the PA outcomes were detected (Table 7). Due to the small sample size for some of the breed groups, only changes over time were examined.

### 3.6. Changes in PA after a Dog Died

At T1 24, T2 16 and T3 17 participants reported that at least one dog had died, respectively. As a consequence, 57 dogs died during the course of the study. However, in the subgroup of participants whose dog died, no significant changes in total PA (Δ = 1.08 ± 12.3, *t* = (0.66, *df* = 56, *p* = 0.510) or leisure time walking (Δ = (0.12 ± 4.7, *t* = 0.20, *df* = 56, *p* = 0.843) were detected, after the dog had died.

Only one of the participants reported not owning another dog after the dog’s death. Before the dog died this participant reported 10.5 h/week of leisure time walking and 16.7 h/week of total PA per week. All leisure time walking was performed as dog walking and dog-related PA accounted for 11.7 h/week (70.1%) of total PA. After the dog died, the participant reported 3 h/week of total PA. This corresponds to a decrease of 82% in total PA. No leisure time walking was reported after the death of the dog.

## 4. Discussion

The main purpose of the present study was to examine the influence of dog size, energy level and dog age on their owners’ PA behavior. At baseline, no statistical group differences were identified for total PA and leisure time walking. In contrast, groups differed significantly in dog-related PA and dog walking as well as in the types of chosen activities.

According to the LMM analysis, total PA, dog-related PA, leisure time walking and dog walking decreased significantly over 3 years in all groups except for leisure time walking in owners of ROTT. In this group, leisure time walking increased over time. In the group of dog owners that completed the trial, no changes in total PA, dog-related PA, leisure time walking and dog walking could be observed.

These findings suggest that the type of PA—and as a potential consequence the intensity of PA—might have a greater impact on physical health of the owners of different dog breeds than just the duration of PA.

The duration and variability in the data of total and dog-related PA is slightly higher than in other German cross sectional studies that used the same questionnaire [6,17]. The reasons for this finding remain unclear. However, high individual variability is a well-known phenomenon in this field of study (e.g., [1,13,15,64,65]).

Old age of dogs is negatively correlated with PA of their owners [35,36,39,40,42]. Thus, it was hypothesized that PA levels of dog owners decrease over time. However, the results only partly support this hypothesis. Although a negative trend was found in the overall population, this trend was not supported if only completers were analyzed. The lack of PA decline could be explained by the fact that many of the dogs might not have been sufficiently old to display an age-related decline in PA. Given the results of Patronek et al. (1997), the mean physiological age of all dog breeds would have been at the younger end of the middle-aged spectrum (28 to 39 human years). Therefore, three years later, the dogs’ mean physiological age would not exceed 55 human years. At T3, the dogs were probably not old enough and the dogs were still too healthy to cause a decrease in their owners’ PA. Another explanation could be that non-completers reported higher amounts of all PA outcomes at T0 and T1 and in total PA at T2. Although not all of the comparisons were significant on a statistical level, the decreases in PA that were detected in the LMMs might be derived from participants who either overreported their PA or were more physically active, but did not complete the study and thus might bias the results. Thorpe et al. reported that in their population, dog walkers’ PA levels decreased at the same rate as in all other groups after three years. However, the participants of Thorpe et al. were between 70 and 79 years old at baseline and, thus, not comparable to the population of the current investigation [66].

Earlier studies demonstrated that having multiple dogs deters dog owners from engaging in dog-related PA [36,67,68]. However, having multiple dogs might also help owners to remain active when one of the dogs gets old or sick. Only one participant reported that her dog died and that no other dog remained in the household afterwards. The level of PA dropped dramatically after the dogs’ death. However, this is only a single case and cannot be extrapolated to a larger group of dog owners. Degeling and Rock report a similar case. They state that one of their participants was less motivated to exercise after the dog’s death, but another participant reported the opposite [40]. In the present study, except for the one named case, there was always another dog living in the household when another dog had died. In these cases, no changes emerged in total PA and leisure time walking when a dog died. This indicates that, if at least one dog remains in the household, the death of one dog does not impact the PA behavior of the dog owner. Future studies are warranted to investigate the relationship of owning several dogs, dog death and PA of the owners.

The results do not show a clear pattern that owners of larger or more energetic dogs were more active than owners of dog breeds that are smaller or less energetic. This contradicts earlier findings [29,31,32,34,36]. It indicates that just the size and energy level of a dog breed are insufficient to predict how much PA the owner will engage in with and without their dog. It suggests that other factors need to be taken into account. However, the cited studies asked the owners for their perception of their dogs. The current study categorized the dog breeds based on their energy level a priori. Consequently, the energy level attributed to each dog by the owner may not match the category based on the breed-average C-BARQ scores. It is possible that the owner’s perception of an individual dog’s energy level may be more reliable than a level derived from averaging multiple assessments of dogs of the same breed.

Further, a cultural element may complicate the interpretation of the influence of a dog’s energy level on the owners’ PA. Nagasawa et al. found that dogs in Japan are perceived as more energetic and restless than dogs in the USA [69]. Therefore, the influence of the energy level of a dog on the PA behavior of their owner might differ between people from different cultural backgrounds. The current investigation used data from the C-BARQ study that takes place in the USA and is mainly performed in English [45]. Thus, it remains unclear whether the average perception of German and US-American dog owners of their dog’s energy level match or whether there are slightly different.

Several differences in the selection of PA types were found between the owners of the different dog breeds. However, due to the limitations of study design, these differences cannot be explained. It could be assumed that some dog breeds are better suited for certain activities than others. Some dogs might not be able to engage in PA at an intensity that is beneficial to the owner. This could explain the lower volume of dog-related PA in CKCS compared to WHWT, LAB, BC, ROTT and BSD. Since no statistically significant differences between the owners of CKCS and owners of the other dog breeds were detected in total PA, this suggests that owners of CKCS engage in other non-dog-related activities more than other owners. This could in turn lead to greater health benefits for the owners of CKCS, due to increased intensities. This may be especially true since an earlier study indicated that dog-related PA are mostly not of a moderate intensity [17]. However, this study did not investigate the types of PA. Therefore, it is not possible to conclusively assess the quality of the non-dog-related PAs.

Some activities might also be performed with certain dog types more often. For example, (rally)obedience was mostly performed by owners of medium to large breed dogs, especially owners of BC, ROTT and BSD. Arhant et al. report that owners of larger dogs are more likely to be engaged in this activity [34]. Especially, owners of ROTT and BSD might perform these activities because they might be afraid that their dogs are strong enough to harm other people and need to be “under the control” of the owner. On the other hand, ROTT do not show increased stranger-directed, dog-directed and owner-directed aggression or dog rivalry as compared to other dog breeds [37]. This could indicate that ROTT owners either successfully take part in activities like (rally)obedience or dog school training. However, the reasons why certain dog owners engage in certain activities remain not fully understood.

There were great differences between the dog breeds in regards to neuter status. Especially JRT/PRT and ROTT were often neutered, while BERN, CKCS and WHIP were more often non-neutered. At the outset, this was not anticipated and the authors have no explanation for this finding. However, it is conceivable that there are owner beliefs about dogs of the selected breeds that have not been surveyed and might influence whether or not owners decide to neuter their dogs.

During the COVID-19 pandemic no serious decrease of PA was detected in this study. Earlier studies that focused on PA during the COVID-19 pandemic identified dramatic declines in moderate to vigorous PA [70,71] with potentially serious health effects [72]. Similar declines in dog walking and PA have been found in some [73,74] but not all [75] studies that focus on dog owners. Thus, the current study indicates that dog ownership could be a protective factor against the decline of PA during the pandemic and that dog owners might benefit greatly from their dogs in terms of PA during the COVID-19 pandemic. Still, it has to be emphasized that the legal framework varied greatly between different countries in regard to the lockdowns. The opportunities owners had to walk their dogs during lockdown varied greatly between different countries. For example, the lockdown in Spain and Serbia included dog walking [73,74], while leisure time walking was allowed during the lockdown in Germany [76]. Overall, it must be considered that the COVID-19 pandemic is an exceptional event that impacts the lives of people worldwide. Therefore, the study results are probably not generalizable, or only with limitations, to a time outside the pandemic.

Overall, this study has some limitations. As with most studies in this field, it relies exclusively on self-reported PA. Several studies in different populations show that over-reporting is a common problem in self-reported PA, especially moderate to vigorous PA [77,78,79,80,81,82]. This may also be true for this study. However, the results are similar to earlier studies that also used the BSA-F [6,17]. Thus, it is likely that the results are reliable.

Further, the BSA-F does not include an assessment of the intensities of PAs. Therefore, it remains unclear whether the intensity of the reported PAs is sufficient to produce health enhancing effects. Overall, results on the intensity of dog-related PA remain controversial. Hielscher et al. considered it likely that most of the dog-related PA failed to achieve moderate intensity [17], which would be necessary to reach the PA guidelines as specified by the World Health Organization (WHO) [83]. However, Richards et al. state that a considerable amount of dog-related PA is of at least moderate intensity [18]. Thus, dog-related PA could be considered to be health enhancing. Furthermore, recent studies highlight the positive impact of light intensity PA on health and mortality [23,84,85,86,87], even though moderate to vigorous PA is considered to be more effective [23,86,87]. Thus, the high levels of PA in this study show that dog owners are likely to benefit from their dogs due to increased levels of PA, regardless of the breed.

The recruitment design of the study was based solely on self-selection in a convenience sample. This might have biased the results because only the most motivated dog owners participated in the study. It is possible that the PA behavior of these owners differs systematically from owners who did not participate in the study. However, self-selection bias is a phenomenon that is not limited to online research, as the results of Oswald et al. show [88]. Nevertheless, interpreting and generalizing the data has to be treated with caution.

Participants were mostly recruited online. It is possible that dog owners who use dog-centric online media are more active with their dog than dog owners who are not organized in dog-related social media groups. This could be related to the fact that dog owners in dog-related online groups identify more strongly with their dog and the ownership of a dog and therefore have different attitudes than dog owners who are not organized in this way, which, in turn, might be reflected in their dog ownership behavior. This, together with self-selection bias, may limit the extent to which the findings can be generalized to the whole dog owner population.

The dropout rate in this study was high. It has been shown that a higher dropout rate is associated with a greater bias in statistical models [89,90]. The results of the completer analysis show that participants with a lower educational status dropped out of the current study more often. This is congruent with the results of Gustavson et al. [90]. This suggests caution when generalizing the current findings. Because the reasons for dropping out of the study could not be investigated, it remains unclear how this could bias the results. However, the fact that completers and non-completers differed in several ways suggests that the results may be biased in some way. Most participants who dropped out terminated their participation in the second year, thus, before the COVID-19 pandemic. Therefore, the authors do not believe that the pandemic influenced the decision to terminate participation to a great extent.

## 5. Conclusions

Overall, the study shows that the PA behavior of owners of the selected breeds is stable over time in this population. The aging of the dog was only found to have a minor influence on the PA of the owners. Anecdotal evidence suggests that losing one’s dog might have a significant, negative impact on dog owners’ PA.

The results also provide evidence that owners of different dog breeds differ in their choice of PA types, as in the duration of total PA, total dog-related PA, leisure time walking and dog walking. The extent to which this influences the health of the dog owner remains unclear and must be examined in future studies.

## Figures and Tables

**Table 1:** Sociodemographics of the participants split by owned breed at T0.

Variable	Manifestation	More than 1 Breed	CKCS	WHWT	JRT/ PRT	WHIP	LAB	BC	BERN	ROTT	BSD
Smoking status	Yes n (%)	5 (38.5)	8 (25.0)	10 (50.0)	7 (24.1)	7 (20.6)	15 (19.5)	30 (30.6)	7 (24.1)	14 (51.9)	30 (40.0)
	No n (%)	8 (61.5)	24 (75.0)	10 (50.0)	22 (75.9)	27 (79.4)	62 (80.5)	68 (69.4)	22 (75.9)	13 (48.1)	45 (60.0)
Education	No degree n (%)	1 (7.7)	0	0	0	0	0	0	0	1 (3.7)	1 (1.3)
	Secondary modern school qualification n (%)	1 (7.7)	2 (6.3)	3 (15.0)	3 (10.3)	0	1 (1.3)	6 (6.1)	1 (3.4)	5 (18.5)	1 (1.3)
	Intermediate high school certificate n (%)	7 (53.8)	12 (37.5)	10 (50.0)	9 (31.0)	9 (26.5)	22 (28.6)	36 (36.7)	12 (41.4)	16 (59.3)	37 (49.3)
	University of applied science qualification or high school diploma n (%)	2 (15.4)	10 (31.3)	5 (25.0)	8 (27.6)	11 (32.4)	21 (27.3)	29 (29.6)	10 (34.5)	3 (11.1)	16 (21.3)
	College or university degree n (%)	2 (15.4)	7 (21.9)	2 (10.0)	6 (20.7)	12 (35.3)	24 (31.2)	26 (26.5)	6 (20.7)	2 (7.4)	20 (26.7)
	Dissertation n (%)	0	1 (3.1)	0	3 (10.3)	2 (5.9)	9 (11.7)	1 (1.0)	0	0	0
Income in €	<1000 n (%)	3 (30.0)	5 (20.0)	2 (10.5)	2 (8.7)	3 (12.5)	5 (8.8)	8 (9.3)	1 (4.5)	3 (12.5)	12 (16.9)
	1000–1999 n (%)	4 (40.0)	12 (48.0)	5 (26.3)	5 (21.7)	2 (8.3)	14 (24.6)	38 (44.2)	7 (31.8)	10 (41.7)	23 (32.4)
	2000–2999 n (%)	2 (20.0)	4 (16.0)	6 (31.6)	7 (30.4)	6 (25.0)	13 (22.8)	22 (25.6)	4 (18.2)	6 (25.0)	13 (18.3)
	3000–3999 n (%)	0	3 (12.0)	3 (15.8)	8 (34.8)	8 (33.3)	9 (15.8)	12 (14.0)	5 (22.7)	1 (4.2)	12 (16.9)
	4000–5999 n (%)	1 (10.0)	1 (4.0)	3 (15.8)	1 (4.3)	5 (20.8)	8 (14.0)	5 (5.8)	4 (18.2)	1 (4.2)	10 (14.1)
	6000–7999 n (%)	0	0	0	0	0	5 (8.8)	1 (1.2)	0	1 (4.2)	0
	8000–9999 n (%)	0	0	0	0	0	2 (3.5)	0	0	1 (4.2)	0
	≥10,000 n (%)	0	0	0	0	0	1 (1.8)	0	1 (4.5)	1 (4.2)	1 (1.4)
Children under 18 years living in the household	Yes n (%)	6 (46.2)	11 (35.5)	1 (5.0)	4 (13.8)	4 (11.8)	16 (21.1)	20 (20.6)	4 (13.8)	8 (29.6)	18 (24.0)
	No n (%)	7 (53.8)	20 (64.5)	19 (95.0)	25 (86.2)	30 (88.2)	60 (78.9)	77 (79.4)	25 (86.2)	8 (70.4)	57 (76.0)
Age of the participant	*M* (*SD*)	37.5 (12.8)	40.8 (11.9)	50.2 (10.9)	44.7 (12.5)	41.2 (12.1)	46.3 (12.2)	39.3 (11.1)	44.1 (12.2)	42.4 (14.5)	43.3 (10.7)
Number of dogs owned	*M* (*SD*)	2.6 (1.0)	2.1 (1.2)	1.6 (1.1)	1.8 (0.9)	2.1 (0.8)	2.0 (1.3)	2.2 (1.1)	1.8 (1.1)	1.5 (.8)	2.4 (1.1)

Notes: Only statistically significant differences are depicted. BC, Border Collie; BERN, Bernese Mountain Dog; BSD, Belgian Shepherd Dog; CKCS, Cavalier King Charles Spaniel; JRT/PRT, Jack and Parson Russell Terrier; LAB, Labrador Retriever; PA, physical activity; ROTT, Rottweiler; WHIP, Whippet; WHWT, West Highland White Terrier.

**Table 2:** Characteristics of the different dog breeds at baseline.

Variable	Manifestation	Other Breed/Mix	CKCS	WHWT	JRT/PRT	WHIP	LAB	BC	BMD	ROTT	BSD	Statistics	*p*
												(*df*)	
Sex	Male	84	23	18	27	43	60	87	21	13	50	18.51 ^a^	0.03
	n	(48)	(41.8)	(58.1)	(52.9)	(69.4)	(42.6)	(47.5)	(48.8)	(39.4)	(41.7)	(9)	
	(%)												
	Female	91	32	13	24	19	81	96	22	20	70		
	n	(52)	(58.2)	(41.9)	(47.1)	(30.6)	(57.4)	(52.5)	(51.2)	(60.6)	(58.3)		
	(%)												
Neutering status	Neutered	97	12	15	31	10	29	50	8	17	29	96.32 ^a^	<0.001
	n	(55.4)	(21.8)	(48.4)	(59.6)	(16.1)	(20.6)	(27.3)	(18.6)	(51.5)	(24.2)	(9)	
	(%)												
	Intact	78	43	16	21	52	112	133	35	16	91		
	n	(44.6)	(78.2)	(51.6)	(40.4)	(83.9)	(79.4)	(72.7)	(81.4)	(48.5)	(75.8)		
	(%)												
Chronic diseases of dogs	Yes	36	11	4	5	6	20	18	4	5	13	14.53 ^a^	0.105
	n	(20.7)	(20)	(12.9)	(9.6)	(9.8)	(14.2)	(9.8)	(9.3)	(15.2)	(10.8)	(9)	
	(%)												
	No	138	44	27	47	55	121	165	39	28	107		
	n	(79.3)	(80)	(87.1)	(90.4)	(90.2)	(85.8)	(90.2)	(90.7)	(84.8)	(89.2)		
	(%)												
Age in years	*M*	6.6	4.3	5.3	6.5	4.5	5.2	4.4	3.6	4.4	4.8	6.36 ^b^	<0.001
	(*SD*)	(4)	(3)	(3.1)	(4.2)	(3.2)	(3.7)	(3.7)	(2.7)	(2.9)	(3.5)	(9, 878)	

Notes: ^a^, *χ*^2^-statistics; ^b^, *F*-value for ANOVA; BC, Border Collie; BERN, Bernese Mountain Dog; BSD, Belgian Shepherd Dog; CKCS, Cavalier King Charles Spaniel; JRT/PRT, Jack and Parson Russell Terrier; LAB, Labrador Retriever; PA, physical activity; ROTT, Rottweiler; WHIP, Whippet; WHWT, West Highland White Terrier.

**Table 3:** Participation of owners of different dog breeds in different types of activities.

Type of Exercise	CKCS	WHWT	JRT/PRT	WHIP	LAB	BC	BMD	ROTT	BSD	*χ*^2^-Value	*p*
	n	T	T	n	n	n	n	n	n	(*df*)	
		n	n								
	(%)	(%)	(%)	(%)	(%)	(%)	(%)	(%)	(%)		
Riding the bicycle with the dog	6	3	7	14	17	26	5	4	26	13.06	0.11
	(18.8)	(15)	(24.1)	(41.2)	(22.1)	(26.5)	(17.2)	(14.3)	(34.7)	(8)	
Ball work	3	8	9	5	9	13	4	5	19	18.58	0.017
	(9.4)	(40)	(31)	(14.7)	(11.7)	(13.3)	(13.8)	(17.9)	(25.3)	(8)	
Jogging	3	0	4	3	11	21	6	4	20	14.35	0.073
	(9.4)		(13.8)	(8.8)	(14.3)	(21.4)	(20.7)	(14.3)	(26.7)	(8)	
(Rally)Obedience	0	0	3	0	4	24	4	9	28	56.6	<0.001
			(10.3)		(5.2)	(24.5)	(13.8)	(32.1)	(37.3)	(8)	
Agility	2	0	4	6	3	40	2	1	9	65.85	<0.001
	(6.3)		(13.8)	(17.6)	(3.9)	(40.8)	(6.9)	(3.6)	(12)	(8)	

Notes: Only participants who participated in the mentioned activity are displayed. Only activities that were mentioned at least 20 times are displayed; BC, Border Collie; BERN, Bernese Mountain Dog; BSD, Belgian Shepherd Dog; CKCS, Cavalier King Charles Spaniel; JRT/PRT, Jack and Parson Russell Terrier; LAB, Labrador Retriever; ROTT, Rottweiler; WHIP, Whippet; WHWT, West Highland White Terrier.

**Table 4:** Linear mixed models of total PA, dog-related PA, leisure time walking and dog walking over time in hours per week.

Outcome	Predictor	Estimate	*SE*	95% CI	*p*
Total PA in h/week	Intercept	26.39	2.53	21.42, 31.37	<0.001
	Linear trend over time per month	−0.09	0.03	−0.15, −0.02	0.009
	CKCS	Ref.			
	WHWT	0.14	4.13	−7.99, 8.27	0.973
	JRT/PRT	−3.58	3.7	−10.85, 3.70	0.334
	WHIP	−5.82	3.52	−12.74, 1.11	0.099
	LAB	−0.63	3.01	−6.54, 5.28	0.834
	BC	−0.12	2.92	−5.86, 5.62	0.966
	BMD	−2.24	3.55	−9.21, 4.74	0.529
	ROTT	6.99	3.69	−0.26, 14.24	0.059
	BSD	2.76	3.04	−3.21, 8.74	0.364
Dog-related PA in h/week	Intercept	11.05	1.36	8.37, 13.73	<0.001
	Linear trend over time per month	−0.05	0.02	−0.08, −0.02	<0.001
	CKCS	Ref.			
	WHWT	4.85	2.24	0.45, 9.24	0.031
	JRT/PRT	0.56	2	−3.37, 4.50	0.778
	WHIP	0.16	1.91	−3.60, 3.91	0.935
	LAB	3.99	1.63	0.80, 7.19	0.015
	BC	4.34	1.58	1.24, 7.44	0.006
	BMD	2.19	1.89	−1.53, 5.91	0.248
	ROTT	6.00	2.00	2.07, 9.92	0.003
	BSD	5.63	1.64	2.40, 8.85	0.001
Leisure time walking in h/week	Intercept	10.56	1.19	8.22, 12.91	<0.001
	Linear trend over time per month	−0.1	0.05	−0.20, 0.00	0.048
	CKCS	Ref.			
	WHWT	3.81	1.95	−0.02, 7.64	0.051
	JRT/PRT	−0.38	1.75	−3.81, 3.05	0.828
	WHIP	−0.51	1.67	−3.79, 2.78	0.762
	LAB	2.06	1.43	−0.74, 4.86	0.15
	BC	1.14	1.38	−1.57, 3.85	0.41
	BMD	0.53	1.69	−2.79, 3.85	0.755
	ROTT	3.12	1.75	−0.32, 6.56	0.076
	BSD	1.98	1.43	−0.84, 4.80	0.168
	CKCS*Linear trend over time per month	Ref.			
	WHWT*Linear trend over time per month	0.01	0.08	−0.15, 0.17	0.898
	JRT/PRT*Linear trend over time per month	0.04	0.07	−0.09, 0.17	0.532
	WHIP*Linear trend over time per month	0.03	0.06	−0.10, 0.15	0.673
	Lab*Linear trend over time per month	0.04	0.06	−0.07, 0.15	0.502
	BC*Linear trend over time per month	0.09	0.06	−0.02, 0.20	0.124
	BMD*Linear trend over time per month	0.10	0.07	−0.04, 0.23	0.153
	ROTT*Linear trend over time per month	0.27	0.07	0.12, 0.41	<0.001
	BSD*Linear trend over time per month	0.05	0.06	−0.07, 0.17	0.407
Dog walking in h/week	Intercept	9.35	1.11	7.17, 11.53	<0.001
	Linear trend over time per month	−0.11	0.04	−0.18, −0.03	0.006
	Quadratic trend over time per month	0.002	0.001	0.000, 0.004	0.036
	CKCS	Ref.			
	WHWT	4.28	1.82	0.71, 7.84	0.019
	JRT/PRT	−0.12	1.63	−3.32, 3.07	0.94
	WHIP	0	1.55	−3.05, 3.04	0.998
	LAB	2.74	1.32	0.14, 5.34	0.039
	BC	2.01	1.28	−0.51, 4.53	0.117
	BMD	1.16	1.55	−1.87, 4.20	0.452
	ROTT	4.22	1.62	1.04, 7.41	0.01
	BSD	2.52	1.33	−0.10, 5.14	0.059

Notes: BC, Border Collie; BERN, Bernese Mountain Dog; BSD, Belgian Shepherd Dog; CKCS, Cavalier King Charles Spaniel; JRT/PRT, Jack and Parson Russell Terrier; LAB, Labrador Retriever; PA, physical activity; ROTT, Rottweiler; WHIP, Whippet; WHWT, West Highland White Terrier.

**Table 5:** Sociodemographic status of completers vs. non-completers.

Variable	Manifestation	Completer	Non-Completer
Gender	Male n (%)	4 (7.7)	48 (92.3)
	Female n (%)	80 (20.9)	302 (79.1)
Smoking	Yes n (%)	16 (12.0)	117 (88.0)
	No n (%)	68 (22.6)	233 (77.4)
Educational attainment	No degree n (%)	0	3 (100.0)
	Secondary modern school qualification n (%)	2 (8.7)	21 (91.3)
	Intermediate high school certificate n (%)	23 (13.5)	147 (86.5)
	University of applied science qualification or high school diploma n (%)	25 (21.7)	90 (78.3)
	College or university degree n (%)	25 (23.4)	82 (76.6)
	Dissertation n (%)	9 (56.3)	7 (43.8)
Employment status	Full time n (%)	42 (19.5)	173 (80.5)
	Part time n (%)	34 (26.0)	97 (74.0)
	Not employed n (%)	8 (9.2)	79 (90.8)
Income in €	<1000 n (%)	3 (6.8)	41 (93.2)
	1000–1999 n (%)	16 (13.3)	104 (86.7)
	2000–2999 n (%)	22 (26.5)	61 (73.5)
	3000–3999 n (%)	16 (26.2)	45 (73.8)
	4000–5999 n (%)	9 (23.1)	30 (76.9)
	6000–7999 n (%)	4 (57.1)	3 (42.9)
	8000–9999 n (%)	0	3 (100.0)
	≥10,000 n (%)	2 (50.0)	2 (50.0)
Chronic diseases of participants	Yes n (%)	12 (10.3)	104 (89.7)
	No n (%)	72 (23.3)	237 (76.7)

Notes: Only statistically significant differences are depicted.

**Table 6:** Differences in PA between completers and non-completers.

Time	Variable	Completer		Non-Completer		*t*	*p*	Cohens *d*
						(*df*)		
		*M* (*SD*)	n	*M* (*SD*)	n			
T0	Total PA in h/week	22.9	84	27.5	351	2.84	0.005	0.29
		(12.3)		(16.4)		(161.5)		
	drPA in h/week	13.3	84	15	351	2.04	0.043	0.2
		(6.3)		(8.9)		(172.6)		
	Leisure time walking in h/week	11	84	12.3	351	1.8	0.074	0.18
		(5.4)		(7.5)		(166.8)		
	Dog walking in h/week	10.4	84	11.5	351	1.62	0.107	0.16
		(5.3)		(7.2)		(162.8)		
T1	Total PA in h/week	21.3	84	26.4	110	2.23	0.027	0.3
		(11.7)		(19.8)		(181.7)		
	drPA in h/week	12.5	84	13	110	0.43	0.665	0.06
		(6.9)		(9.3)		(192)		
	Leisure time walking in h/week	10.3	84	10.8	110	0.47	0.642	0.07
		(5.8)		(7.6)		(192)		
	Dog walking in h/week	9.7	84	10.1	110	0.44	0.658	0.06
		(5.8)		(7.5)		(192)		
T2	Total PA in h/week	21.6	84	23.8	40	0.85	0.397	0.16
		(11.7)		(16.5)		(122)		
	drPA in h/week	13	84	11.7	40	−1.04	0.302	−0.2
		(6.4)		(7)		(122)		
	Leisure time walking in h/week	10.9	84	9.6	40	−1.17	0.243	−0.23
		(5.3)		(6.2)		(122)		
	Dog walking in h/week	10.2	84	9	40	−1.15	0.251	−0.24
		(4.9)		(5.9)		(122)		

Notes: PA, physical activity; drPA, dog-related PA.

**Table 7:** Changes in PA and walking behavior of participants who completed the study.

		T0	T1	T2	T3			
Variable	*n*	*M*	*M*	*M*	*M*	*F*	*p*	Partial
		(*SD*)	(*SD*)	(*SD*)	(*SD*)	(*df*)		*h^2^*
Total PA in h/week	84	22.9	21.3	21.6	21.4	0.86	0.464	0.01
		(12.3)	(11.7)	(11.7)	(14.8)	(3, 249)		
drPA in h/week	84	13.3	12.5	13	12.2	1.45	0.229	0.02
		(6.3)	(6.9)	(6.4)	(6.4)	(3, 249)		
Leisure time walking in h/week	84	11	10.3	10.9	10.5	0.61	0.611	0.01
		(5.4)	(5.8)	(5.3)	(6.3)	(3, 249)		
Dog walking in h/week	84	10.4	9.7	10.2	10.1	0.67	0.572	0.01
		(5.3)	(5.8)	(4.9)	(6)	(3, 249)		

Notes: PA, physical activity; drPA, dog related PA.

## Data Availability

The data on sociodemographics and physical activity are available upon request from Benedikt Hielscher-Zdzieblik, while the data on the C-BARQ are available upon request from James Serpell.

## Supplementary Materials

The following supporting information can be downloaded at: https://www.mdpi.com/article/10.3390/ani12101314/s1, the questionnaire translated into English and used in this study.
