# Supplementary material for: Impact of Dog’s Age and Breed on Dog Owner’s Physical Activity: A German Longitudinal Study
Source: Animals (Basel). 2022 May 20;12(10):1314. doi: 10.3390/ani12101314 (PMC9137655; doi:10.3390/ani12101314)
Supplement: Supplementary file 1 [file animals-12-01314-s001.zip › animals-1661480-supplementary.pdf]

English Translation of the Questionnaire used in Hielscher et al. (2022). Impact of Dog’s Age and Breed on Dog Owner’s Physical Activity: A German Longitudinal Study

Page 1 - Appeal

Welcome to our longitudinal analysis of your physical activity behavior.

First, you will receive the declaration of informed consent. By giving your consent you confirm that your data can be used by the German Sport University Cologne for scientific use only. Please read the following Declaration of Consent very carefully.

After this you will be guided through the questionnaire automatically. The questionnaire asks for your living conditions, the health status of you and your dog and your dog-related and non-dog-related physical activity behavior.

Please keep in mind that there are no wrong answers! If possible, answer spontaneously, as you believe it to be true.

You must be at least 18 years old to participate in the study. Furthermore, you have to own at least one of the following dog breeds: Belgian Shepherd Dog, Bernese Mountain Dog, Border Collie, Cavalier King Charles Spaniel, Labrador Retriever, Parson or Jack Russell Terrier, West Highland White Terrier or Whippet. If you own more than one of the selected dog breeds participation is also possible. Ownership of other dog breeds does not exclude you from this study. **However, if you own six or more dogs you cannot participate in the study.**

If your dog or one of your dogs has or developed a chronic condition or died during the course of the study, this does not exclude you from further participation. This way, we can draw conclusions about the causal relationship of your dog ownership status and your physical activity behavior.

We are pleased that you have decided to participate in the study and thank you in advance for your cooperation.

Page 2 – Declaration of Consent

Explanation

The title of the project is: “Longitudinal Analysis of Physical Activity in Dog Owners in Dependence of the Age of the Dog”.

The project manager in charge is:

Benedikt Hielscher
German Sport University Cologne
Institute of Movement Therapy and Movement-oriented Prevention and Rehabilitation
Am Sportpark Müngersdorf 6
50933 Cologne
(Further, a cell phone number and the e-mail-address were given.)

Our project focusses on the physical activity behavior of dog owners. The main question is whether there are differences between owners of different breeds of dogs. Furthermore, the question is what influence the age of a dog (and related problems) has on the duration and type of physical activity of the owners.

Your task as a participant is to complete the questionnaire as precisely as possible. We will ask you to complete the questionnaire again in one, two and three years in order to find changes in your physical activity behavior.

You have the right to ask questions at any time and to stop the participation at any time without any disadvantages for you.

The German Sport University Cologne has not taken out any subject insurance for this project.

Liability for property damage against the German Sport University Cologne and its employees is excluded, unless the damage incurred is due to intent or gross negligence.

<u>Consent to the processing of personal data</u>

Processing of your personal data in the context of the above-mentioned research project is only possible with your explicit and voluntary consent.

You hereby expressly consent to the German Sport University Cologne collecting, storing and using the following personal data from you for the purpose of the research project: e-mail address, date of birth with initials, biometric data (height and weight), socioeconomic data (e.g. income, level of education), data on exercise behavior with and without a dog.

Data with which it is possible to assign you personally will be deleted after the study.

Your data will only be published in anonymized form, i.e. without the possibility of drawing any conclusions about your person. Your data will also be completely anonymized if it is passed on to third parties. If data is passed on, this is done exclusively for scientific purposes.

You can revoke this consent at any time with effect for the future without disadvantages. In this case, your personal data will be deleted immediately. The revocation of consent does not affect the lawfulness of the processing carried out on the basis of the consent until the revocation. (Further) participation in the research project is only possible if consent is given.

By participating, you confirm that you have voluntarily decided to take part in this study. Have you understood the above statements and do you consent to the above data being used for the above purposes?

☐ Yes ☐ No

Page 3

1. Please provide your email address so we can contact you at upcoming survey times.	______________________________
2. Please give a personal code.<br>The code will provide us with the The code is used at the end of the study to track whether subjects completed the questionnaire at all study time points. This is important because only then we will be able to draw a full picture of the study. Your code consists of your initials and your 8-digit date of birth.<br><br>Example:<br>Johannes Müller, born on 20th Mai 1987.<br>The code would be: JM20051987	Code:_________________________
3. How often did you complete the questionnaire yet?	☐ First time<br>☐ Second time (1 year later)<br>☐ Third time (2 years later)<br>☐ Fourth time (3 years later)

Page 4 – Sociodemography I

1. How old are you? *(only at T0)*	____________________
2. Please give your gender. *(only at T0)*	☐ male<br>☐ female
3. Please provide your height.	Height in meter ____________
4. Please provide your weight.	Weight in kg _________
5. Do you smoke?	☐ Yes<br>☐ No
6. What is your highest educational attainment? *(only at T0)*	☐ No degree<br>☐ Secondary Modern School Qualification<br>☐ Intermediate High School Certificate<br>☐ University of Applied Sciences Qualification or High School Diploma<br>☐ College or University Degree<br>☐ Dissertation
7. What is your net monthly income? (Please enter your total household income here, minus all taxes, insurance and other expenses that you need directly to live). *(only at T0)*	☐ < 1,000 € per month<br>☐ 1,000 - 1,999 € per month<br>☐ 2,000 - 2,999 € per month<br>☐ 3,000 - 3,999 € per month<br>☐ 4,000 - 5,999 € per month<br>☐ 6,000 - 7,999 € per month<br>☐ 8,000 - 9,999 € per month<br>☐ 10,000 or more € per month<br>☐ I do not want to give information.

8. What is your current relationship status?	☐ Single<br>☐ In a relationship, not married<br>☐ Married<br>☐ Widowed<br>☐ Divorced
9. Do children under the age of 18 years live in your household?	☐ Yes<br>☐ No
10. Do elderly people over the age of 59 years live in your household? (Please klick also “yes”, if you yourself are over 59 years of age.)	☐ Yes<br>☐ No
11. Are you employed?	☐ Yes, full time.<br>☐ Yes, part time.<br>☐ No.
12. How many inhabitants does your place of residence have?	☐ less than 5,000 people<br>☐ 5,000 - 19,999 people<br>☐ 20,000 - 99,999 people<br>☐ 100,000 - 499,999 people<br>☐ 500,000 or more people
13. Do you own a garden or are you able to use one?	☐ Yes<br>☐ No
14. Do you suffer from at least one chronic condition that affects your physical activity?	☐ Yes<br>☐ No<br>☐ I do not want to give an answer.

Page 5 – Sociodemography II

15. Please indicate the breed(s) of dog(s) with which you are (first) participating / have participated in the study.<br>**Multiple selection is possible.**	☐ Belgian Shepherd Dog<br>☐ Bernese Mountain Dog<br>☐ Border Collie<br>☐ Cavalier King Charles Spaniel<br>☐ Labrador Retriever<br>☐ Parson Russell Terrier<br>☐ Rottweiler<br>☐ West Highland White Terrier<br>☐ Whippet

Page 6 – Data of Dogs I

16. How many dogs do you own right now?	☐ Right now I do not own a dog.<br>☐ 1 dog<br>☐ 2 dogs<br>☐ 3 dogs<br>☐ 4 dogs<br>☐ 5 dogs

Page 7 – Data of Dogs II

17. Did something change in regard of your dog ownership status? (Did one die, did you give a dog away or did you acquire another dog?)	☐ Yes<br>☐ No

Page 8 – Data of the Dogs III

18. What did change in your ownership status?<br>(Please only give information about long enduring changes.)<br>*(only if participant indicated that there were changes in regard of the ownership status)*	☐ at least one dog died<br>☐ at least one dog was given away<br>☐ at least one dog was acquired<br>☐ other: _______________

Page 9 - 13 – Data of the Dogs IV - VIII (The following data were collected per dog regardless of breed.)

19. Please enter the appropriate data in each of the fields below.	
If your dog is a mixed breed, please simply enter "Mix".	Dog breed: _______________
Please enter the approximate age of your dog in years (e.g. 6 months = 0.5 years).	Age of the dog: ________________
Please enter the approximate height of your dog in centimeters measured at the highest point of the shoulder when standing.	Size in cm: ________________
Please indicate the approximate body weight of your dog in kilo-grams.	Weight in kg: ________________
20. Sex of the dog	☐ Male<br>☐ Female
21. Neutering status	☐ Neutered<br>☐ Not neutered
22. Does your dog have any chronic diseases?	☐ Yes<br>☐ No

Page 14 - Physical Activity, Exercise and Sport Questionnaire (Bewegungs- and Sportaktivitätsfragebogen [BSA-F]) by Fuchs et al. (2015) - Introduction

The following part of the questionnaire is about your physical activity behavior. If you have not performed certain activities, please enter "0" days and "0" minutes.

First of all, your movement activity in your everyday working life is queried. This also includes school, apprenticeship and study. For the individual statements, please click on the category that most closely corresponds to your workplace. If you reported to be unemployed, these questions will not be displayed.

Subsequently, individual activities from your everyday life will be asked. Please enter all of your daily activities that you perform in your free time. If you are not employed/educated, this includes all your of your daily physical activities. Please also indicate activities with your dog, e.g. walks.

The third part refers specifically to dog ownership. Please indicate there once again **only** the activities that you perform **together with your dog**.

In the second as well as in the third part the past 4 weeks (28 days) are asked. Please indicate on how many days you have performed the activity in the past 4 weeks (28 days) and how long this activity has lasted per day you have performed the activity.

Page 15 – Physical Activity, Exercise and Sport Questionnaire (Bewegungs- and Sportaktivitätsfragebogen [BSA-F]) by Fuchs et al. (2015) – Work-Related Physical Activity

Your employment or your training includes… *(not used in the current study)*				
23. seated activities	☐ none	☐ rather little	☐ rather more	☐ much
24. moderate movements	☐ none	☐ rather little	☐ rather more	☐ much
25. intensive movements	☐ none	☐ rather little	☐ rather more	☐ much

(For the original questionnaire please see: https://www.sport.uni-freiburg.de/de/institut/Arbeitsbereiche/psychologie/messinstrumente/Messung%20der%20Sport%20und%20Bewegungsaktivitaet)

Page 16 – Physical Activity, Exercise and Sport Questionnaire (Bewegungs- and Sportaktivitätsfragebogen [BSA-F]) by Fuchs et al. (2015) – Free Time Physical Activity

On how many days and how long did you engage in the following activities **during the last 4 weeks**?

26. Walking to the work-place (also part of the route)	on …… days during the last 4 weeks	ca. …… minutes per day
27. Walking for shopping	on …… days during the last 4 weeks	ca. …… minutes per day
28. Biking to the work-place	on …… days during the last 4 weeks	ca. …… minutes per day
29. Biking for other transportation rea-sons	on …… days during the last 4 weeks	ca. …… minutes per day
30. Going for a walk	on …… days during the last 4 weeks	ca. …… minutes per day
31. Gardening (e.g., mowing the lawn, trimming hedges)	on …… days during the last 4 weeks	ca. …… minutes per day
32. Strenuous home-work (e.g., cleaning, tidying)	on …… days during the last 4 weeks	ca. …… minutes per day
33. Strenuous care (e.g., care for the elderly)	on …… days during the last 4 weeks	ca. …… minutes per day

On how many days and how long did you engage in the following activities **during the last 4 weeks**? *(not used in the current study)*

34. Climbing stairs	on …… days during the last 4 weeks	ca. …… floors per day

(For the original questionnaire please see: https://www.sport.uni-freiburg.de/de/institut/Arbeits-bereiche/psychologie/messinstrumente/Messung%20der%20Sport%20und%20Bewegungsaktivi-taet)

Page 17 – Physical Activity, Exercise and Sport Questionnaire (Bewegungs- and Sportaktivitätsfragebogen [BSA-F]) by Fuchs et al. (2015) – Exercise or Sport related Physical Activity

35. Did you engage in regular exercise or sport activities **during the last 4 weeks?**

☐ Yes ☐ No

36. What kind of exercise or sport activities did you perform?

A) ____________________	I performed this activity during the **last 4 weeks**<br><br>about ..… times,<br><br>and each time for<br>about …… minutes
B) ____________________	I performed this activity during the **last 4 weeks**<br><br>about ..… times,<br><br>and each time for<br>about …… minutes
C) ____________________	I performed this activity during the **last 4 weeks**<br><br>about ..… times,<br><br>and each time for<br>about …… minutes

(For the original questionnaire please see: https://www.sport.uni-freiburg.de/de/institut/Arbeits-bereiche/psychologie/messinstrumente/Messung%20der%20Sport%20und%20Bewegungsaktivi-taet)

Page 18 – Dog-Related Physical Activity I

Please indicate in the following part only the activities you have performed with at least one of your own dogs.

Page 19 – Dog-Related Physical Activity II

37. How often did you walk your dog in the past 4 weeks?	on …… days during the last 4 weeks	ca. …… minutes per day
38. How often did you ride your bike with your dog in the last 4 weeks?	on …… days during the last 4 weeks	ca. …… minutes per day
39. Have you done any other physical activities with your dog in the last 4 weeks? (This includes agility, retrieving, ball work, pulling games, catching games and others).	☐ Yes<br>☐ No	

Page 20 – Dog-Related Physical Activity III

40. Please indicate what other activity or activities you have done together with your dog, how often and for how long in the last 4 weeks.

A) ____________________

I performed this activity during the **last 4 weeks**

about ..… times,

and each time for

about …… minutes.

B) ____________________

I performed this activity during the **last 4 weeks**

about ..… times,

and each time for

about …… minutes.

C) ____________________

I performed this activity during the **last 4 weeks**

about ..… times,

and each time for

about …… minutes.

D) ____________________

I performed this activity during the **last 4 weeks**

about ..… times,

and each time for

about …… minutes.

E) ______________________

I performed this activity during the **last 4 weeks**

about ..… times,

and each time for

about …… minutes.

Page 21 – Further Suggestions

41. Do you have any suggestions or recommendations that you would consider important? You can name general suggestions here, as well as things that relate specifically to your dog.	______________________________<br>______________________________<br>______________________________<br>______________________________<br>______________________________<br>______________________________<br>______________________________

Page 22

42. Did you complete the questionnaire alone?	☐ Yes<br>☐ No
43. Have you completed the questionnaire accurately and to the best of your knowledge?	☐ Yes<br>☐ No

Page 23 – Expression of Gratitude to the Participants

Thank you very much for your participation. We hope that you will also participate in the survey again next year. If something changes in your dog ownership status by then, that is no problem and we would be happy if you would answer the questions again then.

If you have any questions until then you can contact Benedikt Hielscher by e-mail. Or you can contact him by phone at the number: ……. .

Until then, we remain with best wishes and we wish you a successful, healthy year and much joy with your dog.
